# Self-compatibility in peach [*Prunus persica* (L.) Batsch]: patterns of diversity surrounding the *S*-locus and analysis of SFB alleles

**DOI:** 10.1038/s41438-020-00392-z

**Published:** 2020-10-01

**Authors:** Donia Abdallah, Ghada Baraket, Veronica Perez, Amel Salhi Hannachi, Jose I. Hormaza

**Affiliations:** 1 Faculté des Sciences de Tunis, Département Biologie, Université de Tunis El Manar, 2092 Tunis, Tunisie; 2 Laboratorio de Agrobiología Juan José Bravo Rodríguez (Cabildo Insular de La Palma), Unidad Técnica del Instituto de Productos Naturales y Agrobiología (IPNA-CSIC), 38700 S/C La Palma, Canary Islands, Spain; 3 Instituto de Hortofruticultura Subtropical y Mediterránea La Mayora (IHSM La Mayora-UMA-CSIC), 29750 Algarrobo-Costa, Malaga Spain

**Keywords:** Evolutionary biology, Self incompatability

## Abstract

Self-incompatibility (SI) to self-compatibility (SC) transition is one of the most frequent and prevalent evolutionary shifts in flowering plants. *Prunus* L. (Rosaceae) is a genus of over 200 species most of which exhibit a Gametophytic SI system. Peach [*Prunus persica* (L.) Batsch; 2*n* = 16] is one of the few exceptions in the genus known to be a fully self-compatible species. However, the evolutionary process of the complete and irreversible loss of SI in peach is not well understood and, in order to fill that gap, in this study 24 peach accessions were analyzed. Pollen tube growth was controlled in self-pollinated flowers to verify their self-compatible phenotypes. The linkage disequilibrium association between alleles at the *S*-locus and linked markers at the end of the sixth linkage group was not significant (*P* > 0.05), except with the closest markers suggesting the absence of a signature of negative frequency dependent selection at the *S*-locus. Analysis of SFB1 and SFB2 protein sequences allowed identifying the absence of some variable and hypervariable domains and the presence of additional α-helices at the C-termini. Molecular and evolutionary analysis of SFB nucleotide sequences showed a signature of purifying selection in SFB2, while the SFB1 seemed to evolve neutrally. Thus, our results show that the SFB2 allele diversified after *P. persica* and *P. dulcis* (almond) divergence, a period which is characterized by an important bottleneck, while SFB1 diversified at a transition time between the bottleneck and population expansion.

## Introduction

A common observation in plants is the adoption, during evolution, of several strategies to prevent selfing, and thus, promote outcrossing limiting the deleterious effects of inbreeding. In fact, the presence of physical barriers between the female and male parts to avoid self-fertilization, such as dichogamy, monoecy, dioecy, or floral heteromorphy, has evolved in both gymnosperms and angiosperms. In addition, the most common way to avoid self-fertilization in angiosperms is self-incompatibility (SI), a system present in more than half of flowering plant species (more than 100 families). This system has been known from at least 1876, when Charles Darwin observed that some plant species were sterile to their own pollen, but fertile when pollinated from pollen of other individuals of the same species. Firstly, those mechanisms were gathered under the term “self-sterility”, then the term “self-incompatibility” (SI) was proposed and defined as “*the inability of a plant producing functional gametes to reproduce when it is self-pollinated*”^1^.

Maintenance of such ancient system requires strong evolutionary benefits to counteract the advantages of selfing, such as a higher reproductive success. Yet, the loss of SI represents perhaps the most frequent shift in Angiosperm evolution^2^. However, once lost, any system of homomorphic SI is extremely difficult to regain^3–5^. The homomorphic SI system is controlled by a single Mendelian locus (the *S*-locus), which is comprised of tightly linked genes determining self-recognition specificities and many accessory genes which are also necessary for the proper function of SI^1,6^.

Several studies have addressed SI to self-compatibility (SC) transitions. In fact, many phylogenetic analyses using macro- and microevolutionary models have been performed to understand the causes and consequences of these evolutionary shifts. The majority of these studies concluded that SC species have emerged from SI species^7^ and a variety of causes of these reversions have been hypothesized. Mutations affecting genes both linked and unlinked to the *S*-locus seem to be the major causes^4^. On the other hand, the loss of SI in natural populations is often associated with the reduction of population size, which leads to the reduction of sexual compatible partners and the number of SI alleles^8,9^. This would tend to reduce gene flow between populations with different mating systems, and may eventually lead to reproductive isolation and speciation^10^. Hence, the relationship between the loss of SI and speciation is of particular interest^11^. Genetic evidence for the relation between loss of SI and speciation has been accumulated in Brassicaceae. For instance, in *Capsella rubella* and *Leavenworthia alabamica* race a4, the loss of SI was associated with both the split from the closely related outcrossers (*Capsella grandiflora* and *Leavenworthia alabamica* race a1, respectively) and a strong genome-wide genetic bottleneck^11–13^. Bottleneck has also played a role in SI breakdown in small founding populations by depletion of genetic diversity^14–16^. Moreover, range expansion, has been postulated as a driving force behind SI loss, as it may favor self-fertilization^16,17^.

*Prunus* L. (Rosaceae), a genus of over 200 species of deciduous and evergreen trees and shrubs with economically important fruit and nut crops^18^, exhibits an RNase-based Gametophytic SI system (GSI). In this system, pollen rejection occurs in the style if the *S-*allele of the haploid pollen matches one of the *S*-alleles present in the diploid pistil. The gene that controls the female function encodes a stylar ribonuclease (*S*-RNase), whereas a pollen specific F-Box gene (SFB) has been identified as the responsible of the pollen function^19^. SI is generally the rule in this genus and most species are partially or fully self-incompatible. However, in several mostly self-incompatible species, SC genotypes are observed and the transition from SI to SC has been attributed to different causes in the different species. This reversion is mostly related to mutations in pistil and pollen *S*-locus determinants^20,21^. For instance, an insertion upstream of the *S*6m-RNase in sour cherry (*P. cerasus*)^22^ and a similar mutation in Japanese plum (*P. salicina*), *S*e-RNase^23^, reduces the *S*-RNase expression levels leading to an insufficient accumulation of *S*-RNase in the pistil which breaks the recognition function^21^. In sweet cherry (*P. avium*)^24^ and Japanese apricot (*P. mume*)^25^, the SC phenotype was associated with indels in the SFB codifying region causing a frame-shift in translation that produces a nonfunctional truncated protein^20^. In apricot (*P. armeniaca*), two different mutations conferring SC, an insertion in the SFBc allele that produces an SFBc truncated protein and a mutation in *S*-locus unlinked factors, also called modifier genes (m), both independently have been shown to cause the loss of pollen-*S* function^21,26^. In almond (*P. amygdalus*), SC has been attributed to an inactive *S*f-RNase protein as a result of an *S*f allele. Nevertheless, a similar *S*fa (active) allele encodes an active *S*f protein. This apparent paradox was resolved by the discovery that *S*fi and *S*fa are epialleles differing by the methylation of a single nucleotide upstream of the coding sequence^27,28^.

An exception to the widespread SI in the genus is peach [*Prunus persica* (L.) Batsch; 2*n* = 16], a fully self-compatible diploid species with no recent whole-genome duplication. SI to SC reversion in peach remains a puzzling issue and few works have addressed this topic. The wild ancestor of cultivated peach remains unknown and it is probably extinct, although closely related species such as *P. davidiana*, *P. kansuensis* and *P. mira* are cultivated in some regions in China^29^. A first question regarding the evolution of SC in peach is if domestication of this crop that took place about 7000 years ago in China^30^ could have been involved in the SC transition. In fact, several thousand years of domestication have produced more than 1000 cultivars of *P. persica* worldwide, with significant phenotypic differences in fruit size, flavor, and flower type^31^. However, Tao et al.^32^ proposed that if human selection pressure for SC had been the main reason behind the reversion to SC, we would also expect many SC selections in other species of the genus with a large history of cultivation, such as almond, cherry and plum, and this is not the case. At the molecular level, the loss of SI in peach is mainly attributed to a deficiency in pollen *S*-gene expression (SFB) codifying a nonfunctional truncated protein. To date, only four SFB alleles have been identified in peach. Earlier studies reported two SFB alleles, SFB1 and SFB2^21,32,33^. Later, two additional alleles, SFB3 and SFB4, have been identified^34–36^. Tao et al.^32^ have suggested a possible pressure of weak selection for SC at the beginning of peach speciation.

The main objective of this study was to understand the mechanism of the SI to SC transition in *P. persica*. For that, twenty-four peach accessions from different origins were used. The first step was to verify the self-compatible phenotype of the used samples through observation of pollen tube growth in self-pollinated flowers. The second step was to elucidate the pattern of genetic diversity based on nine SSR loci surrounding the *S*-locus at the sixth linkage group. The final step was to sequence and analyze the nucleotide and peptide sequences of the SFB alleles of the studied peach genotypes.

## Results

### Pollination tests

**Fig. 1:**
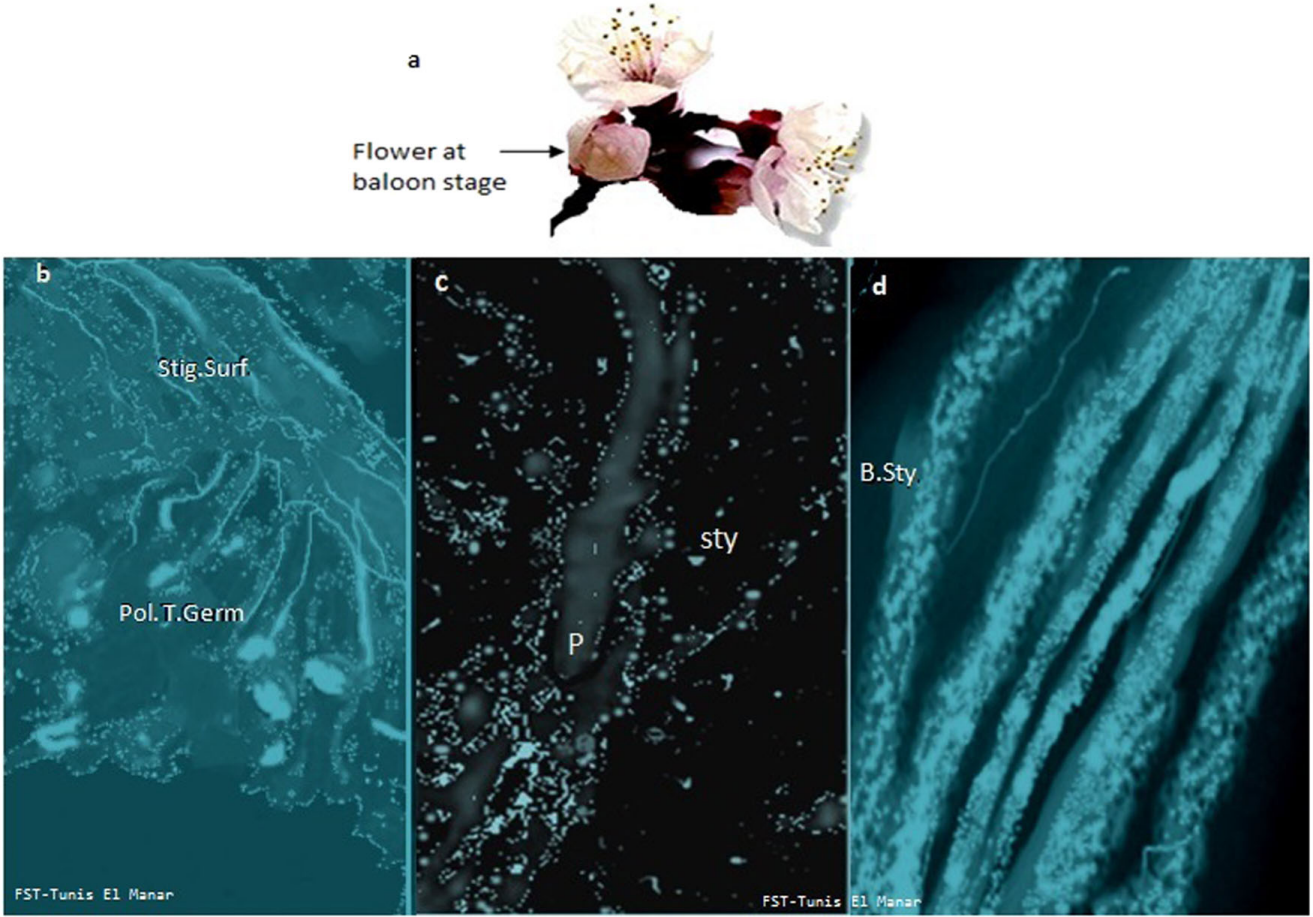
Pollen grain germination and pollen tube growth. **a** The flowers of each genotype were collected at the balloon stage, **b** the pollen grain germination was successful at the surface of stigma in self-pollinated flowers, **c** pollen tube growth was arrested at the style of self-incompatible plum “Cidre” used as reference, and **d** the pollen tubes reached the base of the style in peach and self-compatible plum “Bedri” used as reference. Stig Surf surface of stigma. Pol T Germ pollen tube germination. Sty style. P pollen tube. B Sty base of style. Scale bars = 50 µm

Pollination tests for each genotype were carried out on flowers collected at the balloon stage (Fig. 1a). Each flower was self-pollinated and no intercrosses were made. Pollen germination on the stigma was successful in all cultivars tested (Fig. 1b). After germination, the pollen tubes were arrested in the style of self-incompatible plum (Cidre) used as reference (Fig. 1c), while the pollen tubes reached the bases of the styles in all self-pollinated peach flowers as well as in flowers of “Bedri”, a self-compatible plum confirming the SC phenotype of the peach genotypes studied in this work (Fig. 1d).

### Variability patterns around the *S*-locus

#### Genetic diversity

**Fig. 2:**
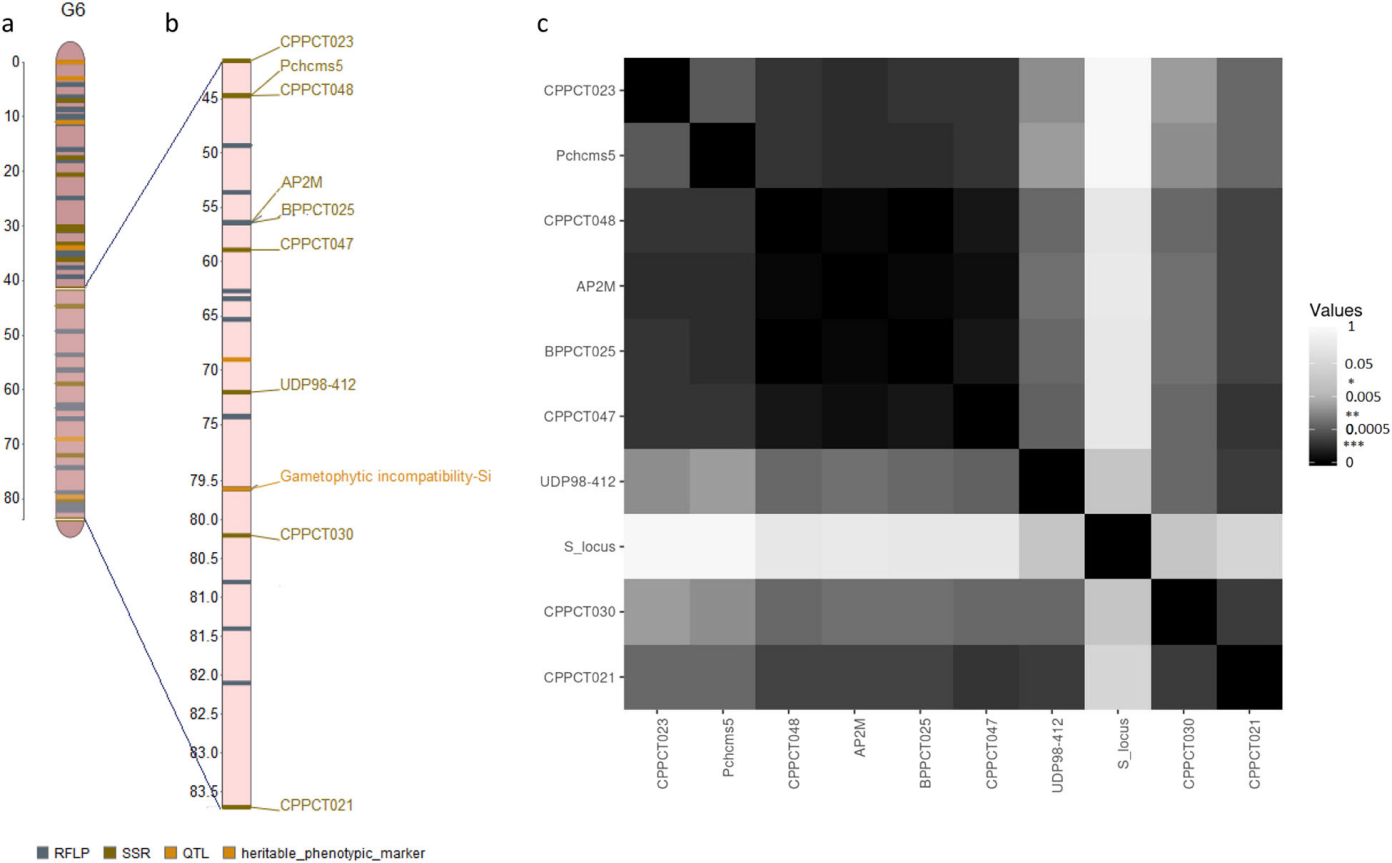
Linkage disequilibrium extent at the end of the sixth LG. **a** Schematic representation of the sixth chromosome in *Prunus*, **b** genetic position of the tested loci mapped in the *Prunus*-TE-F2 linkage map (http://www.rosaceae.org/peach/genome), **c** heatmap representing the distribution of *P* values per pairs of the ten tested loci. *: significance of *P* value

Since in peach, as in other *Prunus* species, the genes directly involved in SI system are clustered at one end of the sixth linkage group^36^, we explored the pattern of diversity along this region using the gametophytic self-incompatibility locus (*S*-locus) and nine SSR markers flanking the *S*-locus mapped in the *Prunus*-TE-F2 linkage map (http://www.rosaceae.org/peach/genome) (Fig. 2a, b).

A total of 39 alleles (*A*o) in the 10 loci studied were amplified in the 24 peach accessions (Table 1). The observed heterozygote fraction (*H*o = 0.4) was lower than the expected (*H*e = 0.44). The PaCons1F/R2 primer, specific to the second intron of the *S*-RNAse gene, amplified the lowest number of alleles (*A*o = 2). The heterozygote level was very low (*H*o = 0.19) giving a low power of discrimination (Pd = 0.19). The CPPCT023 locus, which is ~38 cM upstream the *S*-locus, showed the highest number of alleles (*A*o = 6) and power of discrimination (Pd = 0.72) (Table 1).

**Table 1:** Characteristics and genetic diversity of the 10 loci amplified in the 24 peach accessions studied in this work

Locus	Loci characteristics		Genetic diversity			
	Genetic position (cM)^a^	References	*A*o	*H*o	*H*e	PD
CPPCT023	41.50	Dirlewanger et al.^85^	6	0.5	0.53	0.72
Pchcms5	44.70	Sosinski et al.^86^	3	0.42	0.39	0.48
CPPCT048	44.70	Dirlewanger et al.^85^	5	0.53	0.49	0.6
AP2M	56.40	Aranzana et al.^87^	4	0.39	0.35	0.57
BPPCT025	56.40	Dirlewanger et al.^85^	3	0.42	0.48	0.47
CPPCT047	58.90	Dirlewanger et al.^85^	5	0.45	0.49	0.61
UDP98-412	72.00	Testolin et al.^88^	4	0.32	0.37	0.52
PaCons1F/R2	79.6	Sonneveld et al.^89^	2	0.19	0.33	0.19
CPPCT030	80.20	Dirlewanger et al.^85^	4	0.39	0.42	0.52
CPPCT021	83.70	Dirlewanger et al.^85^	3	0.45	0.48	0.47
Average	–	–	3.9	0.4	0.44	0.5

*Ao* number of observed alleles, *Ho* observed heterozygosity, *He* expected heterozygosity, *PD* power of discrimination

^a^Genetic position of the SSR loci in centimorgan (cM) in the *Prunus*-TE-F2 linkage map (http://www.rosaceae.org/peach/genome)

#### Linkage disequilibrium analysis

A total of 29.5% of intra-chromosome pair comparisons showed significant LD in the genotypes studied. LD association was calculated by considering the significance of LD blocks between each of the alleles at the first and second loci. A total of 65% of the blocks showed significant LD over the tested loci in the analyzed accessions. The heatmap showed that LD association between pair of alleles at the *S*-locus and those linked was not significant (*P* > 0.05), except with the closest locus UDP98-412 (Fig. 2c).

### SFB allele analysis

#### PCR amplification and sequencing

**Fig. 3:**
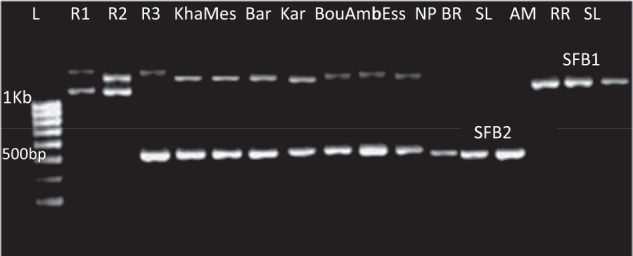
Example of SFB allele amplification in 13 peach samples with PsSFB-F1 and PsSFB-R1. L: 1 KB ladder. R1, R2, and R3: Plum samples used as control with previously known *S-*genotypes. R1 Fortune, R2 Santa Rosa, R3 Beauty. Kha Kharfi, Mes Meski, Bar Bargou, Kar Khoukh Arbi, Bou Boutabgaya, Amb Amber, Ess Essifi, NP Negra Palmera, RM Rojo Mollar, VNZ Venezolano, AM Amarillo Melocoton, RR Rubby Rich, SL Spring Lady

The SFB gene was amplified in the 24 peach accessions. Two bands were obtained, one of 1150 bp and one of 1270 bp (Fig. 3). The obtained fragments were purified and sequenced. Obtained sequences were compared to the data available in Genbank using BlastX and, thus, aligned with the peach SFB1, SFB2, SFB3, and SFB4 alleles. The band of 1270 bp shared more than 98% similarity with the peach SFB1 allele (AB252414) whereas the band of 1150 bp shared more than 98% similarity with the peach SFB2 allele (AB252416).

The SFB alleles obtained in this work were deposited in Genbank under accession numbers KY629929–KY629934 and MN125684–MN125701 (Supplementary Material 1). The SFB genotypes of different samples were identified and detailed in Table 2.

**Table 2:** Origin and *S*-genotypes of the 24 peach accessions analyzed in this work

Origin	Sample	*S-*genotype
Tunisia	Kharfi	S1S2
Tunisia	Meski	S1S2
Tunisia	Bargou	S1S2
Tunisia	Khoukh Arbi	S1S2
Tunisia	Boutabgaya	S1S2
Tunisia	Amber	S1S2
Tunisia	Essifi	S1S2
Tunisia	Bargo limaoui	S2S2
Tunisia	Khoukh ahmer	S1S1
Tunisia	Platine	S2S2
Spain	Amarillo Melocoton	S1S1
Spain	Blanco Mollar	S1S1
Spain	Mollar	S1S1
Spain	Negra Palmera	S2S2
Spain	Venezolano	S2S2
Spain	Rojo Mollar	S2S2
Spain	Amarillo Merollo	S1S2
USA	Rubby Rich	S1S1
USA	Spring Lady	S1S1
USA	Sun Late	S1S2
USA	Fleur De Star	S1S2
USA	Scup	S1S2
USA	Queen Crest	S1S2
USA	Rich May	S1S2

#### Structural features of peach SFB alleles

**Fig. 4:**
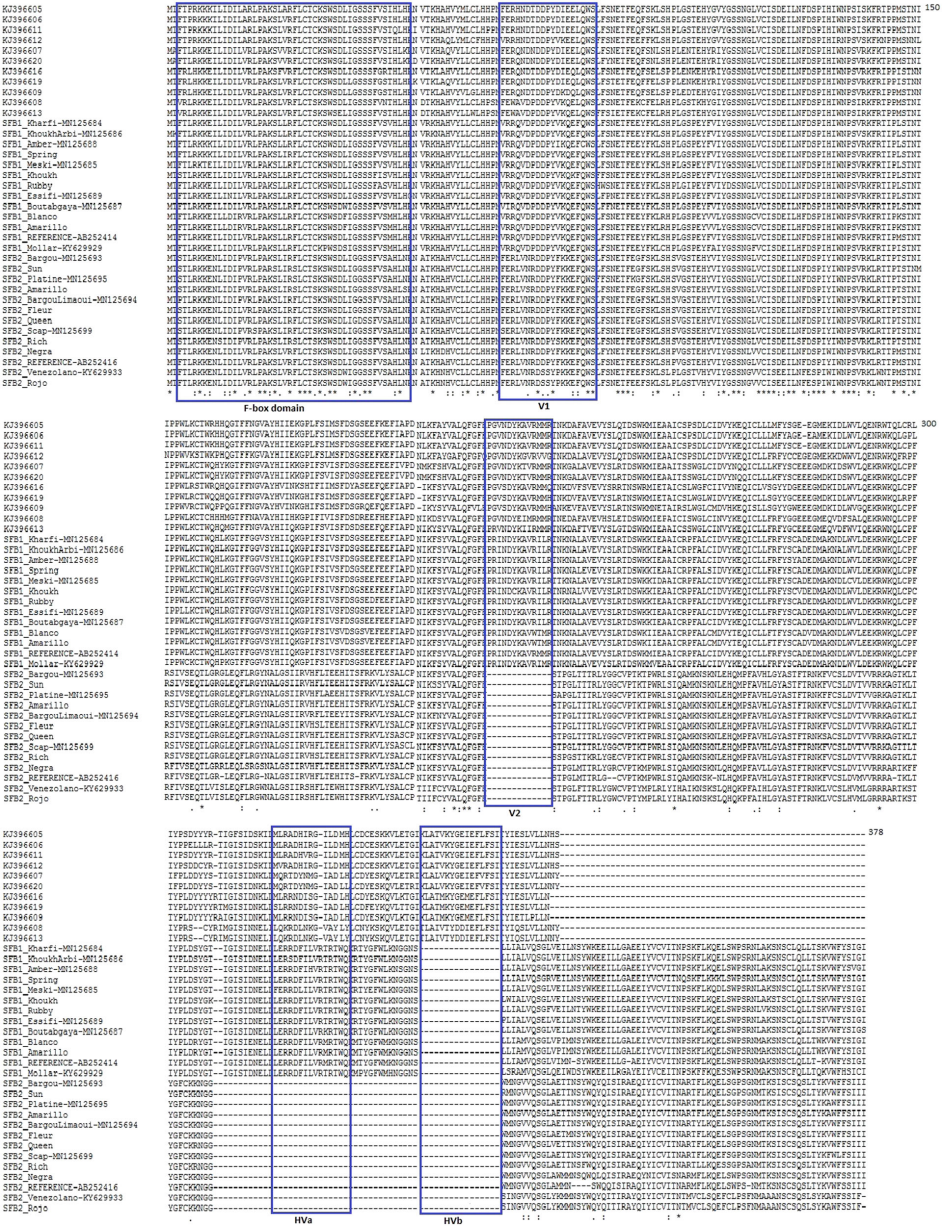
Peptide sequences alignment of peach studied genotypes, two SFB1 and SFB2 reference sequences retrieved from Genebank and 11 Japanese plum SFB sequences. F-box and (hyper) variables regions V1, V2, Hva, and HVb are boxed. The amino acid sequences of SFBs were aligned using Clustal X^73^

To explore the different domains of the SFB *Prunus* gene, we carried out alignments of the deduced amino acid sequences with plum SFB peptides alleles (Fig. 4). The plum SFB peptide sequences showed five domains: the F-box domain localized at the N-termini, two variable regions (designated V1 and V2) located downstream the F-Box, and two hypervariable regions (designated HVa and HVb) at the C-termini^37^. However, the peach SFB1 allele lacked the HVb region whereas the peach SFB2 allele lacked the V2, HVa, and HVb regions (Fig. 4).

**Fig. 5:**
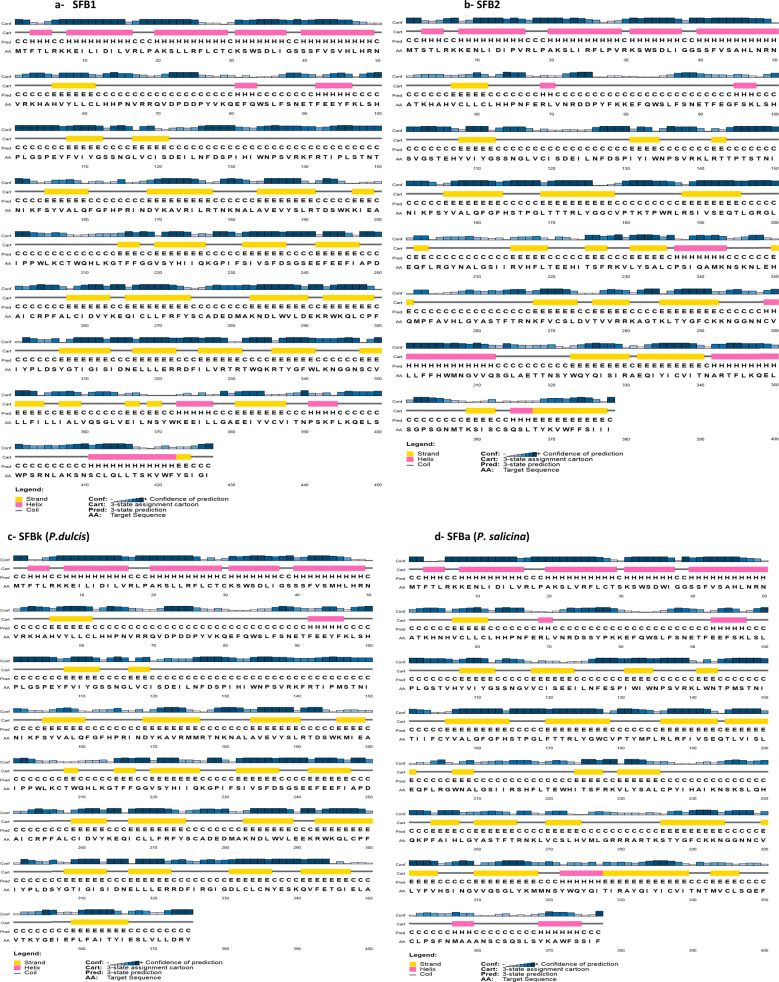
Secondary protein structures predictions of consensus SFB1 sequence (**a**) SFB2 sequence(**b**), SFBk (**c**), and SFBa (**d**). The figures showed the distribution of the β-strands (E), coils and α-helices along the four proteins. Structures were done on the website: http://bioinf.cs.ucl.ac.uk/psipred

Protein secondary structure analyses showed that, overall, the proportion of the β-strands (E) and coils was higher than α-helices in both proteins (Fig. 5). SFB1 protein structure showed 10 α-helices and 23 β-strands (Fig. 5a), while protein structure prediction generated for SFB2 showed 11 α-helices and 19 β-strands (Fig. 5b). The first 60 amino acid fragment showed no differences in the locations and the number of α-helices and β- strands. Variation was observed in the number, nature and position of secondary structural elements from the 70th amino acid. The main difference is related to the presence of additional α-helices in the SFB2 peptide sequence at the 237–243th (α8-helix) and the 299–312nd (α9-helix) amino acid fragments. The C-termini region of both proteins showed considerable differences in the locations and the number of α-helices and β-strands. However, they shared an α-helix conformation at the 411–422nd (α10-helix) fragment in SFB1 and the 342–350th (α10-helix) fragment in SFB2.

In addition, the obtained structures were compared to peptide sequences of self-incompatible almond (SFBk) and self-incompatible plum (SFBa) (Fig. 5c, d respectively), both considered as the ancestral copies of SFB1 and SFB2 respectively^31^. The first 60 amino acids conserve the same location and number of α-helices and β- strands. After that, differences in number and position of the β- strands between each protein and its ancestral copy were observed. The main difference between SFB2 and SFBa resides in the additional α-helices found in SFB2 (α8-and α9-helices). Moreover, the comparison of the C-termini of the four protein structures showed the presence of the additional α-helices found at SFB1 (α10-helix) and SFB2 (α10-helix).

#### Polymorphism and divergence

##### Polymorphism in SFB alleles

In SFB1, 161 polymorphic sites were identified, of which 112 are singletons and 49 are parsimony informative sites. The nucleotide diversity showed a value of 0.08. In SFB2, 228 polymorphic sites were revealed, of which 95 were identified as singletons and 133 were revealed as parsimony informative sites with a nucleotide diversity value of 0.05 (Table 3).

**Table 3:** Patterns of nucleotide diversity and mutational variation in peach SFB nucleotide sequences

	Nucleotide diversity					Mutations								
	*N*	Poly.S	Sing	Par.Inf	*π*^a^	*M*	Syn.M	N.Syn.M	*π*_S_^a^	*π*_A_^a^	*π*_A_/*π*_S_	*K*_S_^a^	*K*_A_^a^	*K*_A_/*K*_S_
SFB1	13	161	112	49	0.08	175	39	136	0.025	0.027	1	0.023	0.025	1
SFB2	13	228	95	133	0.05	244	58	186	0.028	0.055	2	0.05	0.025	0.5

*N* total number of sequences, *Poly.S* polymorphic sites, *Sing* number of singletons, *Par.Inf* parsimony informative sites, *π* nucleotide diversity, *M* total number of mutations, *Syn.M* number of synonymous mutations, *N.Syn.M* number of non-synonymous mutations, *π*_S_ nucleotide diversity at synonymous sites, *π*_A_ nucleotide diversity at non-synonymous sites, *K*_S_ divergence between sequences at synonymous sites, *K*_A_ divergence between sequences at non-synonymous sites

^a^Estimates with Jukes and Cantor correction

The majority of mutations occurred in non-synonymous positions for both SFB1 and SFB2 (N.Syn.M > Syn.M) (Table 3). In SFB1, nucleotide diversity showed similar values between non-synonymous (*π*_A_ = 0.027) and synonymous (*π*_S_ = 0.025) sites. In contrast, in SFB2, nucleotide diversity was higher at non-synonymous (*π*_A_ = 0.55) than at synonymous (*π*_S_ = 0.028) sites (Table 3).

##### Divergence at SFB alleles

Divergence at non-synonymous and synonymous sites (*K*_A_ and *K*_S_, respectively) provides information about the form of sequence evolution in a giving gene. Thus, pairwise sequence divergences were calculated.

As shown in Table 3, synonymous and non-synonymous sites are evolving at equal rates for SFB1 since *K*_A_/*K*_S_ = 1. In SFB2, divergence at synonymous sites showed a value of *K*_S_ = 0.05, while the divergence at non-synonymous sites showed a value of *K*_A_ = 0.025.

#### Evolutionary implications

##### Neutrality tests: SFB alleles are selectively non-equivalent

Result of the McDonald and Kreitman (MK) test in SFB1 sequences supported the null hypothesis^38^ since the ratio of polymorphic sites at non-synonymous and synonymous sites (*P*_A_/*P*_S_ = 3.4) equaled the number of fixed differences at non-synonymous and synonymous sites (*f*_A_/*f*_S_ = 3.6). However, in SFB2 sequences, the ratio *f*_A_/*f*_S_ was lower than the ratio *P*_A_/*P*_S_ with values of 1.63 and 2.6, respectively (Table 4).

**Table 4:** Summary of neutrality tests

	McDonald–Kreitman test						Tajima			Fu and Li	
	Fixed		Polymorphic		*f*_A_/*f*_S_	*P*_A_/*P*_S_	*D*_T_	*D*_S_	*D*_A_	D*	F*
	*f*_S_	*f*_A_	*P*_S_	*P*_A_							
SFBp1	5	18	34	118	3.6	3.4	−1.71	−1.5	−0.9	−1.7	−2
SFBp2	19	31	53	141	1.63	2.6	−1.15	−0.97	−1.5	−0.45	−0.72

*f*_S_ fixed synonymous substitutions between sequences, *f*_A_ fixed non-synonymous substitutions between sequences, *P*_S_ polymorphic synonymous substitutions between sequences, *P*_A_ polymorphic non-synonymous substitutions between sequences, *D*_T_ Tajima statistic for all sites, *D*_S_ Tajima statistic at synonymous sites, *D*_A_ Tajima statistic at non-synonymous sites. D* and F* Fu and Li statistics

Based on the frequency spectrum of polymorphism, a second class of neutrality tests was conducted. Tajima^39^ *D* and Fu and Li^40,41^ D* and F* statistics showed values of −1.71, −1.7, and −2.0, respectively, in SFB1 and values of −1.15, −0.45, and −0.72, respectively, in SFB2 (Table 4). Tajima *D* statistic was calculated at synonymous and non-synonymous sites separately. In SFB1, a negative *D* value of *D*_A1_ = −0.9 was observed at non-synonymous sites and a higher value of *D*_S1_ = −1.5 was revealed at non-synonymous ones. However, in SFB2, a value of *D*_A2_ = −1.5 was found at non-synonymous sites, while the *D* value at synonymous sites was *D*_S2_ = −0.97 (Table 4).

##### Evolutionary relationships between SFB1 and SFB2 sequences

AUPGMA dendrogram and a Minimum Spanning Network were drawn in order to trace the evolutionary relationships between SFB1 and SFB2 alleles and their ancestral copies. In fact, the SFB1 allele is a pollen part mutant (PPM) related to the almond (*Prunus dulcis*) SFBk allele, while the SFB2 allele is a PPM version related to the Japanese plum (*Prunus salicina*) SFBa allele^31^. Two sequences of SFBk and SFBa were retrieved from GenBank data libraries under accessions numbers AB252408 (SFBk) and AB252410 (SFBa).

**Fig. 6:**
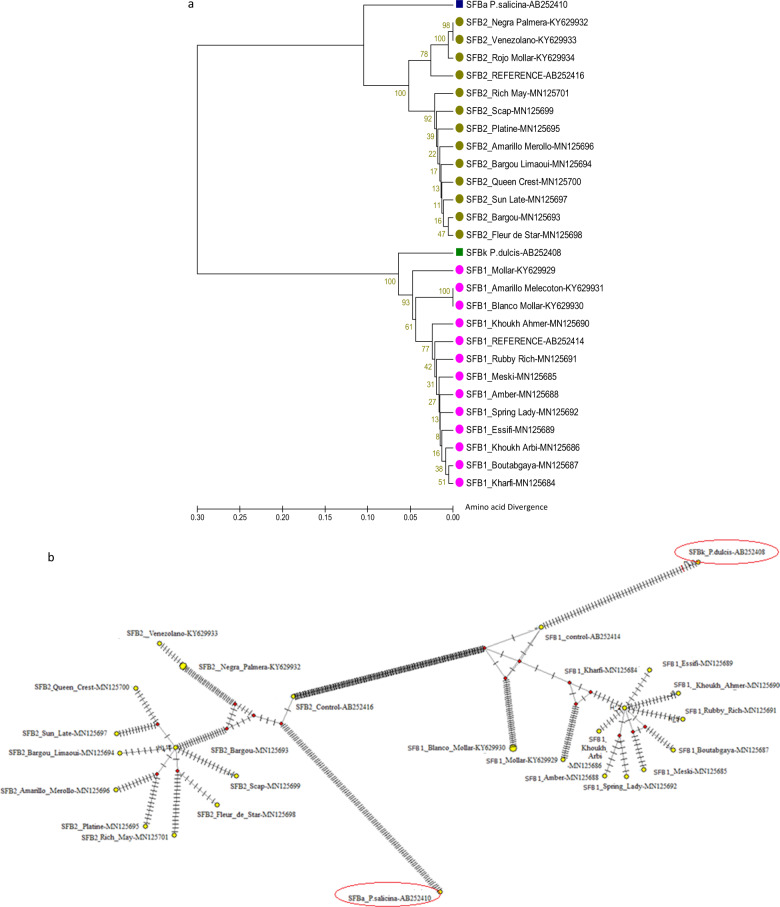
Evolutionary relationships between the SFBp alleles and their ancestral copies. **a** UPGMA tree: the optimal tree with the sum of branch length = 1.16720195 is shown. The percentage of replicate trees in which the associated taxa clustered together in the bootstrap test (500 replicates) are shown next to the branches. The tree is drawn to scale, with branch lengths in the same units as those of the evolutionary distances used to infer the phylogenetic tree. The evolutionary distances were computed using the Poisson correction method and are in the units of the number of amino acid substitutions per site. The analysis involved 28 amino acid sequences. There were a total of 374 positions in the final dataset. **b** Minimum spanning network: the network was constructed using the median joining algorithm. The estimated number of mutations of the shortest tree = 651. The total number of taxa = 28. The total number of haplotypes is 26

The UPGMA tree divided the sequences into two main groups (Fig. 6a). The first gathered the SFB1 and SFBk sequences, while the SFB2 and SFBa sequences fell into the second group. The topology of the dendrogram showed that the divergence between SFBa and SFB2 occurred before the divergence between SFBk and SFB1.

The minimum spanning network confirmed the UPGMA results and showed a higher number of mutational events between the SFB2 and SFBa alleles than between SFB1 and SFBk alleles (Fig. 6b). These mutational events represent the parsimony informative sites described in Table 3.

In order to estimate the approximate age of SFB1 and SFB2 alleles, the rho statistic implemented in the NETWORK software was computed between the ancestral nodes and the descendants’ sequences. The mutational rate used in this work was 10^−3^ mutation per year for GSI *S*-alleles^40^. The approximate age of SFBa compared to all SFB2 sequences was 13,1571.429 years, whereas the age of SFBk compared to all SFB1 sequences obtained was 66,642.857 years. The approximate ages of the oldest SFB peach alleles were estimated by calculating the rho statistic between the closest SFB sequences (SFB1_control and SFB2 control) to the ancestral alleles. The approximate age of SFB1_control compared to SFBk was 25,500 years, whereas the approximate age of SFB2_control was 53,000 years.

## Discussion

In this study, 24 peach cultivars were used to analyze the loss of the SI system in this species. Pollination tests were carried out to confirm the self-compatible phenotype of the studied genotypes. Genetic diversity around the *S*-locus was tested using nine surrounding SSR loci. In addition, we analyzed the SFB alleles, SFB1 and SFB2, since it has been proposed that SC in peach is mainly due to defective SFB alleles^32^.

### Genetic diversity of peach cultivars around the *S*-locus

Linkage disequilibrium (LD) analysis at one end of the sixth peach LG showed nonsignificant LD between the *S*-locus and the surrounding SSR loci. In contrast, significant LD blocks on LG6, particularly between the *S*-locus and closely related SSR markers, were observed in other *Prunus* species such as wild and sweet cherry^42^ as well as in other species in the Bassicaceae such as *Arabidopsis thaliana*, *Arabidopsis lyrata*^43^, *Capsella rubella*^43,44^, and *Biscutella neustriaca*^44,45^. This significant LD is due to the influence of negative frequency dependent selection (NFDS) force in the genomic neighborhood of the *S*-locus. NFDS is a form of balancing selection by which rare alleles escape loss by drift. This selective force can extend the high polymorphism at the selected locus to closely linked neutral sites^46^. Thus, the absence of significant LD between the *S*-locus and closely related SSR markers in *P. persica* species suggests the absence of NFDS forces at the *S*-locus in this species.

### Analysis of the SFB gene in *P. persica*

In this study, two of the four SFB alleles reported so far in peach were revealed in the 24 accessions studied. The absence of SFB3 and SFB4 alleles may confirm that SFB3 may be specific to ornamental cultivars, while SFB4 is restricted to wild genotypes as revealed by Hanada et al.^34^ although other studies such as that of Gu et al.^35^ have reported the presence of SFB3 and SFB4 in cultivated varieties.

On the other hand, the results showed a predominance of S1S2 genotypes in Tunisian cultivars which may confirm previous observations on the presence of a geographical feature of the *S*-genotypes in peach^34,35^. In contrast, in self-incompatible *Prunus* species such plum^47^ and sweet cherry^48^, a lack of correlation between *S*-alleles and the geographic origin of the cultivars was observed indicating that *S*-alleles coalesced before *Prunus* speciation^47^. Thus, *P. persica* SFB alleles seem to have diversified after peach speciation.

A common structure in the SFB peptide sequence in different *Prunus* species is the presence of the F-box and the hypervariable and variable regions^35,49^. The lack of variable and hypervariable regions in SFB peptide sequences confirmed that the SFB peach gene encodes a truncated protein. Similar results were obtained by Gu et al.^35^ where the SFB1 and SFB3 alleles lacked the HVb region, whereas the SFB2 and SFB4 alleles lacked the V2, HVa and HVb regions. In fact, the V2, HVa, and HVb regions are located at the 3′ end of the SFB gene and play a crucial role in the allele-specific recognition function implying that the C-terminal region may be exposed on the surface of the SFB protein, and, thus, be responsible for the discrimination between self and nonself *S*-RNase proteins^36,47^. For this reason, the most obvious structural difference between SFB1, SFB2 and their ancestral versions, involved the presence of α-helices at SFB1 HVb region and SFB2 V2, HVa, and HVb regions. A similar result was observed in five almond genotypes, where differences related to the conformation of secondary structure elements were found at the variable V4 and hypervariable RHV regions^28^. Nevertheless, further studies in peach are required to ascertain whether these structural differences mediate SC.

### Analysis of selective forces at the *P. persica* SFB alleles

The analysis of polymorphism and divergence of the SFB1 allele as well as the MK test (*P*_A_/*P*_S_ ≈ *f*_A_/*f*_S_) suggest that the SFB1 allele seems to evolve neutrally. In fact, the null hypothesis of the MK test implied that the ratio of polymorphic sites at non-synonymous and synonymous sites (*P*_A_/*P*_S_) should equal the number of fixed differences at non-synonymous and synonymous sites (*f*_A_/*f*_S_)^38^. However, the negative values obtained by Tajima *D* statistic and Fu and Li’s D* and F* tests in SFB1 sequences, suggest a recent selective sweep (which removed all the variation in the region), a recent population expansion, and/or an ongoing purifying selection because all tend to produce alleles at low frequency^50^. Previous works^32,48,51^ have denied the notion of a selective sweep in the *Prunus* SFB gene. On the other hand, the higher negative *D* value of Tajima at synonymous than at non-synonymous sites (*D*a1 < *D*s1) observed in SFB1 alleles, confirmed neither population expansion nor purifying selection. In fact, demographic factors, such as a population expansion, equally affect all genes and all regions of a gene which would create a homogeneous effect on all types of mutations and we would expect equal Tajima values of *D* statistic at synonymous and at non-synonymous sites (*D*a < *D*s). However, selective forces, such as purifying selection, directly affect the genetic diversity at a target site and modify the genetic diversity within different sites^52^. Hence, purifying selection has a heterogeneous effect by eliminating deleterious non-synonymous mutations but a little or no effect on neutrally evolving synonymous changes and we would expect a *D* value higher at non-synonymous than at synonymous sites (*D*a > *D*s)^50,52^. This result suggests that the SFB1 allele has diversified from its progenitor (SFBk) in a transition time between two different events, one resulting in a homogenous effect and the other resulting on a heterogeneous effect. In fact, a transition time between homogenous and heterogeneous processes (such population expansion and purifying selection respectively) would result in a period *when neither population expansion nor purifying selection can be detected* as described by Hahn et al.^50^. A similar result was observed by Hahn et al.^50^ in an experimental system of T7 bacteriophage. At the statistical level, the slight difference between mutational classes was due to selective processes and the slight skew at synonymous mutations reflected demographic processes.

In SFB2, the relatively higher non-synonymous polymorphism (*π*_A_ = 0.055) suggested that non-synonymous replacements are more common than synonymous ones giving *π*_A_/*π*_S_ > 1. However, the lower *K*_A_/*K*_S_ ratio suggests that most of the non-synonymous mutations could be deleterious^53^. These results indicate that the deleterious mutations have not been fixed in the SFB sequences and, thus, created a strong non-synonymous nucleotide diversity such noted by Hughes^53^. Afterward, they would have been eliminated by a strong ongoing purifying selection. The signature of purifying selection was confirmed by the MK test (*f*_A_/*f*_S_ < *P*_A_/*P*_S_), suggesting that a high proportion of non-synonymous changes were disadvantageous and, therefore, strongly affected polymorphism. In addition, the increased negative *D* value at non-synonymous sites observed in SFB2 (*D*a > *D*s) revealed a signature of a heterogeneous effect and confirmed the signature of a strong ongoing purifying selection.

### A hypothetical evolutionary scenario for peach self-incompatibility breakdown

In light of the obtained results, SFB2 allele diversified before SFB1, most probably soon after peach speciation. As reported by Ohta^54^, at the time of speciation, the magnitudes of drift may increase and the selective force may change. In particular, after a strong bottleneck, such as that of peach in recent breeding history, the selection coefficients change through space and time^55^. In addition, Tao et al.^32^ and Chen et al.^36^ have reported a selection pressure for SC at the beginning of peach speciation. PPMs might preferentially be selected compared to pistil part mutants due to the higher number of pollen grains produced by each flower^36^. Yet, at the time of SFB2 diversification, purging of deleterious mutations was more efficient because of the smaller population size giving rise to reduced inbreeding depression and/or a decreased *S-*alleles number^13,56,57^.

Due to selfing, we would expect a population expansion after the bottleneck. At the transition time between bottleneck and population expansion, SFB1 would have diversified from SFBk. In parallel, purifying selection would become increasingly efficient in purging deleterious changes getting closer to neutrality.

At the transition between bottleneck and population expansion, the effect of various amino acid changes became effectively neutral. Once diversified, SFB1 alleles behave like neutral alleles. Such status is called “The Nearly-Neutral Theory”^58^, and represented a development of Kimura’s Neutral Theory of molecular evolution. The *Nearly-Neutral Theory* makes testable predictions that go beyond the mere null model of the Neutral Theory i.e., a theory of “no effect”^54^. Most of those predictions strongly support the results reported in this work. One of these predictions is that slightly deleterious variants will accumulate in a species that has undergone a severe bottleneck, and then they will have more chances of being purged by slightly purifying selection than being fixed by positive selection. In fact, unlike strictly neutral alleles, the fate of nearly neutral alleles depends on effective population size. Thus, when the effective population size gets larger for a long time, selection will decrease the frequency of slightly deleterious variants in the population and eventually eliminate them^54^.

### Association between demographic events and self-incompatibility breakdown

One question regarding the SC reversion in peach that remains unclear is whether SI–SC transition occurred before or after peach speciation and the results of this work do not allow solving this question. In fact, it is unclear whether the loss of SI in peach was due to the loss of function mutations (in which case the SC transition should have taken place before peach speciation) or to the impossibility to select a compensatory mutation due to purifying selection (in which case the SC transition should have taken place after peach speciation). Regardless of when the SC reversion took place in peach evolutionary history, our results showed that the loss of SI in peach was the result of an association between mutational events, bottleneck and population expansion.

A similar association between transition from SI to SC and strong bottleneck has been observed in several taxa. For example, in Brassicaceae, the loss of SI in *Capsella rubella* was associated with the split from its SI progenitor species *Capsella grandiflora* (50–100,000 years ago in relation to migration into glacial refuges) and a strong genetic bottleneck^11,13,59,60^. Also, the split between the SI *Leavenworthia alabamica* race a1 and the SC race a4 (150,000 years ago) was associated with a shift from SI to SC, with strong genome-wide genetic bottleneck and evolution of the selfing syndrome^12,13,61^, although it is still unclear whether the SC transition occurred at the time of the split or later^13^.

## Conclusion

During evolution, peach has lost irreversibly the SI system. Our results show that no NFDS takes place at the *S*-locus and confirm a probable purifying selection system in SFB2, while SFB1 seems to evolve neutrally. The loss of the SI system in peach was due to the loss-of-function mutations in the C-terminal regions. In addition, the evolutionary history of *P. persica* played a crucial role to make complete and irreversible the loss of SI.

## Materials and methods

### Plant material

Twenty-four peach accessions from different origins were used in this work (Table 2). Ten of them were collected in Tunisia from growers’ orchards and represent local endemic accessions; seven accessions were developed in the US and were collected from a Tunisian germplasm collection (Sodon); seven accessions were collected from farmer orchards in La Palma Island (Canary Islands, Spain).

### Self-incompatibility phenotype analysis

To verify the SC phenotype of the 24 peach genotypes analyzed, self-pollinations were carried out in the laboratory. Two plum cultivars [“Bedri” (SeSh), known to be self-compatible, and “Cidre” (SaSe) considered as self-incompatible^61^] were used as controls that proof the success of this experimental approach in different species. Flowers of each genotype were collected at the balloon stage and pollen was obtained by manually removing and drying the anthers at room temperature during 24 h. The pollen was then sieved through a fine mesh and stored at −20 °C until required^62–64^. For pollinations, 15–20 flowers of each accession were collected at the balloon stage, 24 h before anthesis, emasculated and maintained on wet florist foam at laboratory temperature^64^. On the following day, the flowers were hand pollinated using a fine paintbrush. Three days later, the pollinated pistils were fixed in FAA [70% ethanol: acetic acid: formaldehyde (18: 1: 1, v/v/v)]^62,63^. For microscope preparations, the fixed pistils were washed three times for 1 h with distilled water and left in 5% sodium sulfite at 4 °C. To soften the tissues, the pistils were autoclaved at 1 kg/cm^2^ during 10 min in sodium sulfite^65^, and stained with 0.1% (v/v) aniline blue in 0.1 N K_3_PO_4_ to stain callose^66^. Pollen tube growth in the style was observed under an Olympus BH2 microscope with UV.

### SSR analysis

#### DNA extraction and amplification

Fresh young leaves were collected from each genotype and frozen. Genomic DNA was extracted using the cetyltrimethyl ammonium bromide (CTAB) method following the protocol described by Doyle and Doyle^67^. Extracted DNA was quantified using a Nanodrop 1000 and diluted to 10 ng/µ*L*.

Ten loci on the sixth linkage group of peach were amplified: the second intron of the *S*-RNase gene (the female GSI component) of the *S*-locus and nine surrounding SSR markers (Table 2). PCR reactions were carried out in a volume of 20 µl, with 20 mMTris–HCl, pH 8.4, 50 mMKCl, 4 mM MgCl2, 0.1 mM of each dNTP, 0.2 μM of each primer, 40 ng of genomic DNA and 0.45 U of BioTaq™ DNA polymerase (Bioline, London, UK). PCR reactions were run in an I-cycler (Bio-Rad Laboratories, Hercules, CA, USA) thermocycler using the following temperature cycles: an initial step of 2 min at 94 °C, 35 cycles of 45 s at 94 °C, 45 s at 57 °C, 1 min at 72 °C, and a final step of 5 min at 72 °C. The obtained fragments were analyzed by capillary electrophoresis with a Beckman Coulter GenomeLabGeXP^TM^ capillary DNA analysis system. Forward primers were labeled with a fluorescent dye on the 5-end. Samples were denaturalized at 90 °C for 120 s, injected at 2.0 kV for 30 s, and separated at 6.0 kV for 35 min.

#### Diversity analyses

To explore the genetic diversity, the number of observed alleles (*A*o), the observed (*H*o) and expected (*H*e) heterozygosities and the power of discrimination (PD) of the *S*-locus and SSR loci were calculated with Arlequin ver. 3.5^68^. LD was estimated by computing squared allele frequency correlations (*r*^2^) between each pair of alleles among the 10 loci with PowerMarker 3.25 software, considering unphased genotype data^69^. Since the distribution of allele frequencies may have an effect on the extent of LD, rare alleles whose frequencies are <5%, were excluded prior to further LD analyse^42^.The distribution of *P* values per pairs of the ten tested loci was represented by heatmap using the Heatmapper.ca online server^70^.

### SFB gene analysis

The SFB gene was amplified using the general primers PsSFB-F_1_ and PsSFB-R_1_^71^ with PCR specifications described in Abdallah et al.^47^. The amplified fragments were separated using a 1.5% agarose gel electrophoresis, stained with SYBRGreen and visualized with UV light. Fragment size estimation was done using a size standard (1 kb DNA Ladder; Invitrogen, Carlsbad, CA, USA). Amplification products were purified using the QIAquick Gel Extraction Kit (Qiagen, Hilden, Germany) and sequenced with a Ready Reaction Big Dye Terminator Cycle Sequencing automated sequencer.

#### Datasets and sequence alignment

Nucleotide sequences were translated to amino acids using the DAMBE program^72^, then DNA and peptide alignments were carried out using the accurate CLUSTALX algorithm version 1.64b^73^ and minor adjustments were performed as described by Hammer et al.^74^. Database searches were performed using the National Center for Biotechnology Information’s: Basic Local Alignment Search Tool (BLAST).

The consensus peptide sequences were obtained using the HIV Sequence Database (http://www.hiv.lanl.gov/).The protein secondary structure predictions were conducted using PSIPRED Version 4.01 (http://bioinf.cs.ucl.ac.uk/psipred)^75,76^.

#### Sequence analysis

Diversity parameters and neutrality tests were calculated using DNAsp 5.1^77^. All alleles were included in the calculations and all sites with alignment gaps were eliminated.

Nucleotide diversity (*π*) was measured as the average number of pairwise nucleotide differences among all sequences^77^. Rates of synonymous and non-synonymous substitutions (*π*_S_ and *π*_A_) in the coding regions were estimated via the approximate method of Nei and Gojobori^78^ with the Jukes and Cantor correction^79^ for multiple hits. The mean ratio of the number of non-synonymous substitutions per non-synonymous site (*K*_A_) to the number of synonymous substitutions per synonymous site (*K*_S_) was also calculated.

The MK test^38^, Tajima statistic (D)^39^, and Fu and Li’s D* and F*^40^ were calculated. In addition, Tajima’s D was calculated in partitioned synonymous and non-synonymous data (*D*_S_ and *D*_A_, respectively).

The evolutionary history was inferred using UPGMA method^80^. The evolutionary distances were computed using the Poisson correction method^81^ and are in the units of the number of amino acid substitutions per site. The analysis involved the amino acid sequences. All ambiguous positions were removed for each sequence pair and evolutionary analyses were conducted in MEGA6^82^.

The Minimum Spanning Network between SFB nucleotide sequences was run into the Network software ver.10.0.0.0using the Median joining algorithm^83^. The rho statistic, estimated by Network, measures the age of an ancestral node in mutational units. This mutational age was then converted into years by multiplication with the mutation rate^84^.

## Supplementary information

Supplementary table 1. Accession numbers and references of nucleotide SFB sequences used in this work
