# Supplementary material for: Self-compatibility in peach [Prunus persica (L.) Batsch]: patterns of diversity surrounding the S-locus and analysis of SFB alleles
Source: Hortic Res. 2020 Oct 1;7:170. doi: 10.1038/s41438-020-00392-z (PMC7527504; doi:10.1038/s41438-020-00392-z)
Supplement: Supplementary file 1 — Supplementary table 1. Accession numbers and references of nucleotide SFB sequences used in this work [file 41438_2020_392_MOESM1_ESM.docx]

| **SFB sequence** | **Species or accession** | **Accession number** | **Reference** |
| --- | --- | --- | --- |
| SFBp1 | Kharfi | MN125684 | This work |
| SFBp1 | Meski | MN125685 | This work |
| SFBp2 | Bargou | MN125693 | This work |
| SFBp1 | Khoukh Arbi | MN125686 | This work |
| SFBp1 | Boutabgaya | MN125687 | This work |
| SFBp1 | Amber | MN125688 | This work |
| SFBp1 | Essifi | MN125689 | This work |
| SFBp2 | Bargo limaoui | MN125694 | This work |
| SFBp1 | Khoukh ahmer | MN125690 | This work |
| SFBp2 | Platine | MN125695 | This work |
| SFBp1 | Amarillo Melocoton | KY629929 | This work |
| SFBp1 | Blanco Mollar | KY629930 | This work |
| SFBp1 | Mollar | KY629931 | This work |
| SFBp2 | Negra Palmera | KY629932 | This work |
| SFBp2 | Venezolano | KY629933 | This work |
| SFBp2 | Rojo Mollar | KY629934 | This work |
| SFBp2 | Amarillo Merollo | MN125696 | This work |
| SFBp1 | Rubby Rich | MN125691 | This work |
| SFBp1 | Spring Lady | MN125692 | This work |
| SFBp2 | Sun Late | MN125697 | This work |
| SFBp2 | Fleur De Star | MN125698 | This work |
| SFBp2 | Scup | MN125699 | This work |
| SFBp2 | Queen Crest | MN125700 | This work |
| SFBp2 | Rich May | MN125701 | This work |
| SFBp1-control | *P.persica* | AB252414 | [31] |
| SFBp2-control | *P.persica* | AB252416 | [31] |
| SFBk | *P.dulcis* | AB252408 | [31] |
| SFBa | *P.salicina* | AB252410 | [31] |
| SFB e | *P.salicina* | KJ396605 | [37] |
| SFB e | *P.salicina* | KJ396606 | [37] |
| SFB b | *P.salicina* | KJ396607 | [37] |
| SFB c | *P.salicina* | KJ396608 | [37] |
| SFB h | *P.salicina* | KJ396609 | [37] |
| SFB e | *P.salicina* | KJ396611 | [37] |
| SFB e | *P.salicina* | KJ396612 | [37] |
| SFB c | *P.salicina* | KJ396613 | [37] |
| SFB h | *P.salicina* | KJ396616 | [37] |
| SFB h | *P.salicina* | KJ396619 | [37] |
| SFB b | *P.salicina* | KJ396620 | [37] |

Supplementary table 1. Accession numbers and references of nucleotide SFB sequences used in this work.
